# Virulence of *Streptococcus mutans*: An intrafamilial cohort study on transmission of genotypes

**DOI:** 10.4317/jced.56038

**Published:** 2020-01-01

**Authors:** Ana-Lídia-Soares Cota, Valter Silva, Salete-Moura-Bonifácio da Silva

**Affiliations:** 1 Postgraduate Program in Society, Technology and Public Policy (SOTEPP), Centro Universitário Tiradentes (UNIT/AL), Maceió (AL), Brazil; 2 Bauru Dental School, Universidade de São Paulo (USP), Bauru (SP), Brazil

## Abstract

**Background:**

The main aims of this cohort study were to measure the intrafamilial risk of transmission, sharing and stability of the most virulent *S. mutans* genotypes.

**Material and Methods:**

A total of 392 clinical isolates of *S. mutans* obtained from caries-active adults and genotyped to evaluate their transmissibility over time. After extraction of the chromosomal DNA, PCR were performed to detect the genes involved in the production of GbpA (gbpA) and mutacin types I, II, III and IV (mutAI, mutAII, mutAIII and mutAIV).

**Results:**

The gbpA, mutAI, mutAII, mutAIII and mutAIV genes were detected in 77.3, 12.5, 51, 16.6 and 89.8% of *S. mutans* isolates, respectively. The virulence of *S. mutans* was associated with its transmission (*P*< 0.01) and stability (*P* = 0.01), with the most virulent genotypes having higher transmissibility (RR = 1.83, 95% CI 1.44 to 2.32) and higher stability in the oral cavity (RR = 1.52, 95% CI 1.06 to 2.19).

**Conclusions:**

Genotypes with the genetic information to synthesize GbpA and mutacins present an important ecological advantage in the process of colonization by *S. mutans*; they remain stable among the oral microbiota of the host and favor intrafamilial transmission.

** Key words:**Streptococcus mutans, virulence factors, transmission, dental caries.

## Introduction

Due to its virulence factors, *Streptococcus mutans* (*S. mutans*) is considered the most cariogenic microorganism to colonize the human oral cavity (1). Consequently, for decades, the scientific community has been dedicated to investigating *S. mutans* using various biochemical, serological and genetic techniques (2).

The virulence of *S. mutans* is related, among other mechanisms, to its ability to synthesize glucan-binding proteins (GBPs), a heterogeneous group of proteins that promote cell adhesion to tooth surfaces (3). GBPs are known to influence the maintenance of the dental biofilm architecture by joining bacteria to extracellular glucan molecules (4), which contribute to plaque formation and to the subsequent development of dental caries (5). GbpA, a GBP that depends on the quantity of environmentally available glucan (6), contributes to *S. mutans* cariogenicity through its role in bacterial adhesion and cohesion during dental biofilm formation (7).

Mutacins also represent an important virulence factor associated with the risk of dental caries. The adsorption capacity of active molecules of mutacins on the surface of sensitive bacteria through specific or not specific receptors can increase their antimicrobial efficiency (8). Furthermore, when a child is exposed to infection by an *S. mutans* strain exhibiting an increased level of mutacin production, it can be presumed that under favorable circumstances the strain will colonize, especially if the ﬂora has not yet reached stability (9).

Previous research (thesis available at: http://dx.doi.org/10.11606/T.25.2007.tde-09112007-094447) revealed that not all *S. mutans* genotypes detected in parents remained sTable over time and colonized the oral cavity of their children. In addition, a systematic review (10) showed that caries risk assessment in children generally is based in case-control or cross-sectional and, cohort studies with adequate follow-up are needed. The aims of this cohort study were 1) to determine the frequencies of genes involved in the production of GbpA (gbpA) and mutacin types I, II, III and IV (mutAI, mutAII, mutAIII and mutAIV) in *S. mutans* and 2) to measure the intrafamilial risk of transmission, sharing and stability of genotypes of virulent *S. mutans* genotypes.

## Material and Methods

-Study Population and Bacterial Isolates

This cohort study used 392 samples of *S. mutans* isolated from the saliva of 20 caries-active adults (with one or more cavitated lesions) and stored at -86°C in brain heart infusion (BHI) broth containing 20% glycerol. The participants were members of eight Brazilian families with low socioeconomic status living in areas with sub-optimal concentrations of fluoride (0.60 to 0.79 mg F/L). All mothers were primiparous and had salivary levels of streptococci from the mutans group ≥ 106 CFU/mL. The isolates had already been identified as *S. mutans* by checkerboard DNA-DNA hybridization and genotyped by arbitrarily primed PCR (AP-PCR) using the arbitrary primer OPA-02, in a previous research (thesis available at: http://dx.doi.org/10.11606/T.25.2007.tde-09112007-094447). Microbiological examinations were performed on participants at baseline (T1) when the eldest son was 7-8 months old and after 22 months (T2) of follow-up. In the present work were used the adults *S. mutans* isolated (mothers, fathers and grandparents) since the genotypes transmitted from parents to children were identical. A pilot study was conducted with a randomized subsample (n = 20) from computer-generated random sets. This research was approved by the Research Ethics Committee of the Bauru School of Dentistry at the University of São Paulo (FOB/USP; Protocol 073/2011).

-Cultivation and Transfer of *S. mutans*

In a laminar flow hood, 10 μL of each thawed and homogenized sample was streaked in Petri dishes (90×15 mm) containing blood agar medium (BioCen do Brasil, Campinas, São Paulo State (SP), Brazil) and on plates (Interlab Distribuidora de Produtos Científicos SA, São Paulo, SP, Brazil) containing mitis salivarius bacitracin sucrose selective medium (11) (Difco Laboratories, Detroit, Michigan (MI), USA). The plates were stored in anaerobic jars (Difco Laboratories, Detroit, MI, USA) and incubated under microaerophilic conditions (candle flame method) at 37°C for 72 hours. After this period, a putative identification of *S. mutans* species was conducted based on the morphology and purity of the microbial culture (11) with a stereo microscope (Olympus, Model SZ 40, Shinjuku-ku, Tokyo, Japan).

A representative colony of *S. mutans* from each mitis salivarius bacitracin sucrose agar plate was selected and aseptically transferred to a culture tube containing 5 mL of BHI broth (Difco Laboratories, Detroit, MI, USA). The tubes were incubated at 37°C in anaerobic jars for 48 hours under microaerophilic conditions. Once bacterial growth was observed, 500 μL was suspended in 20% glycerol and frozen at -80°C. Likewise, aliquots of 1,000 μL were distributed in 2 mL Eppendorf tubes and stored at room temperature for a maximum of 24 hours until the next step was performed.

-Bacterial DNA Extraction

The QIAamp DNA mini kit (Qiagen, Valencia, California (CA), USA) was used for the extraction of bacterial chromosomal DNA following the protocol for manipulating gram-positive bacteria. Sequentially, the purity and concentration of the extracted genetic material were determined by spectrophotometry (NanoDrop spectrophotometer ND-1000, Thermo Fisher Scientific, Wilmington, Delaware, USA). The extract purity was considered adequate when the DNA samples showed an A260 nm/A280 nm ratio between 1.8 and 2.0. Through DNA dilution in ultrapure water, the samples were standardized to a final concentration of 15 ng/μL and stored at -20°C.

-Genetic-Molecular Analysis: GbpA

Bacterial chromosomal DNA samples were amplified by PCR using specific primers flanking the gbpA gene that encodes the GbpA protein (5’-TAGATATCCGACAATTTGCAAGTAATAGAAGT-3’ and 5’-TAGATATCCGTTATCATACGACGACATACAA-3’) (6).

Each PCR mixture, in a reaction volume of 25 μL, consisted of 2.5 μL of 10X buffer (10 mM Tris-HCl, 50 mM KCl, pH 8.0), 0.75 μL of MgCl2 (1.5 mM), 0.5 μL of dNTPs (0.2 mM), 0.2 μL of forward primer (0.4 μM), 0.2 μL of reverse primer (0.4 μM), 0.25 μL of Platinum® Taq DNA polymerase (1.25 U) (Invitrogen Life Technologies, São Paulo, SP, Brazil), 15.6 μL of ultrapure water and 5 μL of bacterial DNA. The PCRs were optimized using a strain *S. mutans* UA159 (ATCC 700610) as a positive control (C+). Additionally, to ensure the absence of contaminating DNA, a sample containing ultrapure water was included as a negative control (C-) in each set of reactions.

The PCRs were performed on a thermocycler (Applied Biosystems, Veriti Model 96, Foster City, CA, USA) with an initial denaturation of the DNA strands at 95°C for 1 minute, followed by 35 subsequent cycles of denaturation at 95°C for 15 seconds, annealing of the primers at 59°C for 30 seconds and extension at 72°C for 30 seconds. The end of the thermocycling occurred with a final extension at 72°C for 7 minutes.

The amplified DNA fragments were separated by 0.75% agarose gel electrophoresis (Invitrogen Life Technologies, São Paulo, SP, Brazil), submerged in 1X TBE (Tris-borate-EDTA, pH 8.0) buffer. The electrophoretic runs were conducted in a horizontal tank (Loccus Biotecnologia, Model LCH 12X14, Cotia, SP, Brazil), maintaining a constant current at 100 V with an electrophoresis power source (Loccus Biotecnologia, Model LPS 300V, Cotia, SP, Brazil). The gels were stained with SYBR Safe™ DNA gel stain (Invitrogen Life Technologies, Eugene, Oregon, USA) and visualized under ultraviolet light using a 250 bp molecular weight marker (DNA Ladder, Invitrogen Life Technologies, St. Paulo, SP, Brazil). Images of the gels were captured, and bands were analyzed by visual comparison, and the presence of the gbpA gene was confirmed by amplifying and visualizing a single band of 2,100 bp.

-Genetic-Molecular Analysis: Mutacins

Bacterial chromosomal DNA samples were amplified by PCR using specific primers flanking the mutA genes that encode mutacin types I and III (5’-AGTTTCAATAGTTACTGTTGC-3’ and 5’-GCCAAACGGAGTTGATCTCGT-3’) (12,13), II (5’-AACGCAGTAGTTTCTTTGAA-3’ and 5’-TTCCGGTAAGTACATAGTGC-3’ (14) and IV (5’-ATGGGATATTTAAAGGGAAA-3’ and 5’-TCAGAGCAGCTACAAAAACT-3’) (15). Only one pair of primers was selected to detect the mutAI and mutAIII genes due to the high homology between them (15).

The PCRs were like genetic-molecular analysis by GbpA. Thermocycling was performed with an initial denaturation protocol at 95°C for 1 minute, followed by 40 cycles of 95°C for 15 seconds, annealing of the primers at 58°C for 30 seconds and extension at 72°C for 30 seconds and a final extension at 72°C for 7 minutes. The DNA amplification products were separated by 1.5% agarose gel electrophoresis, and the presence of the mutAI, mutAII, mutAIII and mutAIV genes was confirmed by bands of 700, 444, 450 and 1,344 bp, respectively.

-Data Analysis and Operational Definition

The data for all the variables and categories were analyzed using descriptive and inferential statistics. The absolute and relative detection frequencies of the gbpA, mutAI, mutAII, mutAIII and mutAIV genes in the clinical isolates of *S. mutans* were assessed. *S. mutans* genotypes were grouped as least (up to two genes detected) or most virulent (three to five genes detected). The chi-square test was used to evaluate the association between the virulence of *S. mutans* isolates and their transmission, sharing and stability. When at least one expected value was < 5, Mid-P exact test was used and 0.5 was added to each cell for calculation. The risk of the transmission, sharing and stability for the most virulent *S. mutans* genotypes was estimated by the relative risk. Etiologic fraction in the population was estimated to assess the proportion for the most virulent *S. mutans* genotypes that would be prevented if the risk factors were eliminated. All estimates were calculated with a 95% confidence interval by Taylor series variance approximation. P-value were reported and was applied a significance level of 5%. Importantly, the genotypes considered sTable were the ones detected in T1 and T2. Due to the occurrence of genotype sharing among family members, the use of the term transmission was restricted to the transfer of microorganisms from adult individuals to children.

## Results

General characteristics at baseline and distribution of number of *S. mutans* isolates from each family member over the periods (baseline and after 22 months of follow-up) are presented in  Table 1.

**Table 1:**
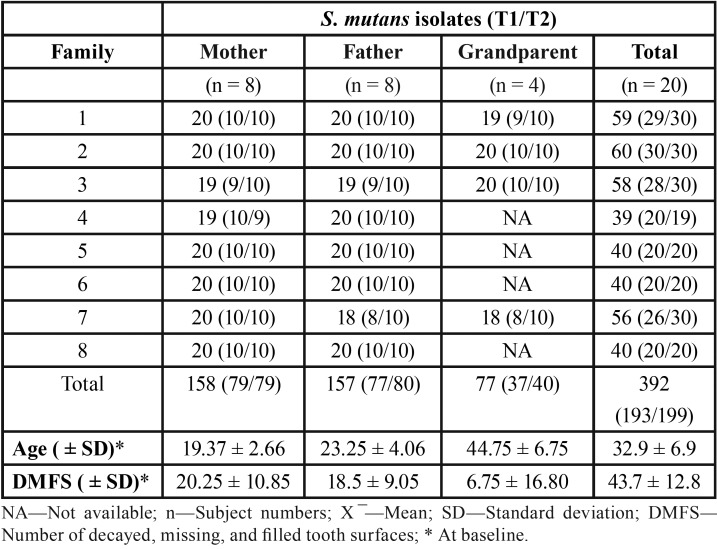
Distribution of number of S. mutans isolates from each family member at baseline (T1) and after 22 months (T2) of follow-up and general characteristics.

The specific primers PCR amplified at least one and at maximum four virulence genes from all of *S. mutans* isolates, corresponding to most and least virulent genotypes isolated. The gbpA, mutAI, mutAII, mutAIII and mutAIV genes were detected in 303/392 (77.3%, 95% CI 72.9 to 81.2), 49/392 (12.5%, 95% CI 9.6 to 16.1), 200/392 (51.0%, 95% CI 46.1 to 55.9), 65/392 (16.6%, CI 95% 13.2 to 20.6) and 352/392 (89.8%, 95% CI 86.4 to 92.4) of isolates, respectively.

The set of clinical isolates used (N = 392) included all 24 different *S. mutans* genotypes previously genotyped and represented by different letters; 11/24 (45.8%) genotypes were identified as the most virulent ( Table 2).  Table 2 also shows the qualitative virulence ratings for all the identified *S. mutans* genotypes and also identifies those transmitted from adults to children in each family, those shared by at least two subjects in a family and those that remained sTable in the oral cavity of at least one family member over 22 months of follow-up. High virulence was observed in 6/11 transmitted genotypes, 10/23 shared genotypes and 8/17 sTable genotypes.

**Table 2:**
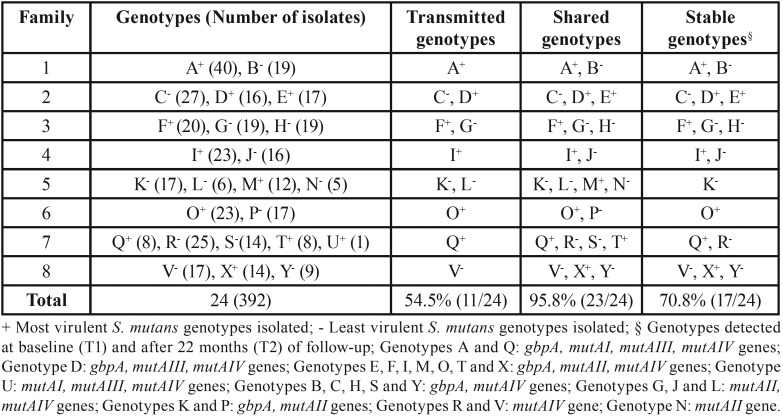
Characterization of *S. mutans* genotypes detected.

The virulence of *S. mutans* was associated with its transmission (*P* < 0.01) and stability (*P* = 0.01), but not with sharing (*P* = 0.96). The most virulent genotypes having higher transmissibility (RR = 1.83, 95% CI 1.44 to 2.32) and higher stability in the oral cavity (RR = 1.52, 95% CI 1.06 to 2.19). With a 95% confidence interval, between 14.7% to 32.2% of transmission of most virulent *S. mutans* genotypes would be prevented if the risk factors were eliminated ( Table 3).

**Table 3:**
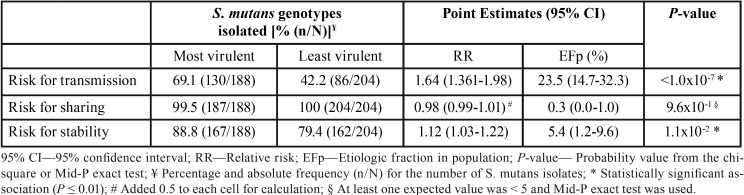
Virulence of *S. mutans* isolates and the transmission, sharing and stability of genotypes.

## Discussion

The present work provides insight into the mechanism by which bacterial virulence contributes to the colonization process of *S. mutans*, which significantly influences its transmission capacity and stability. *S. mutans*, the main pathogen associated with dental caries, expresses several virulence genes that influence its growth and accumulation on the tooth surface. To our knowledge, this is the first cohort study with adequate follow-up to report the presence of the gbpA gene in clinical isolates of *S. mutans*. Previous studies were strictly laboratory based and assessed the clinical significance of GbpA by deleting the gbpA gene in reference strains (4,16).

In the present study, the genes coding for mutacin types IV and I were the most and least prevalent, respectively. However, the mutAI gene was found to be the most prevalent in previous research (17). The low detection frequencies of some of these structural genes reveal the existence of a wide genetic diversity in the mutA gene locus or even its absence in the samples tested (18).

Due to the broad detection spectrum shown by the analyzed genes, we chose to group the *S. mutans* genotypes into different virulence classes. This approach made it possible to identify those genotypes with the genetic potential to function as highly virulent colonizers. The detection of these genotypes in 48% of the analyzed *S. mutans* isolates could indicate an important biological role of their encoded proteins in the formation of dental biofilm. *S. mutans* genotypes that produce a broad spectrum of mutacins tend to become predominant over time in most of the oval cavity sites (19).

There is evidence of vertical transmission of *S. mutans*, i.e., from mother to child. A systematic review and meta-analysis demonstrated an association between *S. mutans* in mothers and their respective children (20). From this study, the possible intrafamilial transmission between the mother-child pairs was also evidenced among the children and their respective parents and grandparents. This intense sharing of genotypes within each family made it difficult to accurately identify the transmission routes and indicates the need for a reassessment of antimicrobial preventive models focused only on the maternal role (e.g., if the *S. mutans* genotype was shared by all family members, the child may have acquired the genotype from the mother, the father or even the grandmother). Our results for etiologic fraction in the population indicates that around 20% to 43% of transmission the most virulent *S. mutans* genotypes might be prevented if the risk factors for transmission were eliminated.

In the present research, the size effect of the relative risks between the virulence of *S. mutans* genotypes and their transmission capacity and stability were essential in determining that the more virulent genotypes had a greater risk of undergoing transmission from adults to children and of persisting temporally. However, the virulence of *S. mutans* was not associated with the degree of genotypic similarity observed among the family members, although the sharing of at least one high-virulence *S. mutans* genotype was recognized in all the studied families. This observation indicates a pattern to share at least one high-virulence strain of *S. mutans*, i.e., lack of sharing is rare.

In contrast with the present study, other researchers (18) found that not all the isolates transmitted from mothers to their respective children carry the mutAI gene. It is necessary to consider that the transmission of *S. mutans* is a process influenced not only by microbial factors but also by host and environmental factors, which modulate host immune defenses and bacterial competitiveness (21). In addition to the salivary level of *S. mutans* (22), transmission fidelity has been associated with the type of delivery (18), the duration and intensity of breastfeeding (23) and the race of the host (24).

The oral cavity is an open growth system. This means that nutrients and microorganisms are repeatedly introduced and removed, and only those that are able to adhere to a surface or otherwise find refuge in the grooves, cracks or interproximal spaces can overcome the removal forces imposed by salivary flow (25). *S. mutans* may derive benefit from the availability of specific adhesion mechanisms, particularly those mediated by GbpA. Since the inactivation of the gbpA gene tends to reduce sucrose-dependent adhesion *in vitro* (26) and *in vivo* (7), its detection in the analyzed bacterial isolates could explain the persistence of the genotypes in the oral cavity of the family members.

There was wide variability in the genetic determinants associated with the detection of *S. mutans* virulence factors. This variability was demonstrated by the broad spectrum of gbpA, mutAI, mutAII, mutAIII and mutAIV genes detected among the samples studied. These genotypes were more likely to be virulent, with increased transmission from adults to children and persistence in the oral cavity. Thus, the present study suggested that *S. mutans* genotypes with the genetic information to synthesize GbpA and mutacins show an important ecological advantage during the colonization process, remaining sTable in the oral microbiota of the host and favoring bacterial transmission among individuals.
